# The prevalence and associated characteristics of Bipolar Disorder diagnosis among admitted patients at three tertiary psychiatric hospitals in Zimbabwe: A cross sectional study

**DOI:** 10.1371/journal.pone.0290560

**Published:** 2024-01-02

**Authors:** Rukudzo Mwamuka, Malinda Kaiyo-Utete, Chido Mawoyo, Walter Mangezi

**Affiliations:** Mental Health Unit, Faculty of Medicine and Health Sciences, University of Zimbabwe, Harare, Zimbabwe; Singapore General Hospital, SINGAPORE

## Abstract

**Background:**

Bipolar Affective Disorder (BD) is a serious condition that affects more than 1% of the world’s population. If not treated can cause disability, yet its prevalence in Zimbabwe is not known. This study explores the burden of Bipolar Disorder and its associated factors in Zimbabwe.

**Methods:**

A cross sectional study with a sample of 272 participants was carried out at three tertiary hospitals in Zimbabwe. Data was collected using an interviewer administered questionnaire and the Mini International Neuropsychiatric Interview (M.I.N.I). The study shows the prevalence and factors associated with Bipolar Disorder at tertiary psychiatric hospitals. Data analysis was done using STATA S/E 13.0 for data management.

**Results:**

The prevalence of BD in the sample was 39.3%. Factors associated with BD were, being formally employed (AOR = 3.69, 95%CI: 1.55–8.79), a history of defaulting medications (AOR = 1.90, 95%CI: 1.02–3.57) and a reported previous diagnosis of BD (AOR = 5.66, 95%CI: 2.72–11.8).

**Conclusions:**

The prevalence of BD among admitted participants in tertiary psychiatric hospitals in Zimbabwe is high. It is comparable to that from African studies done in clinical settings. There is need for in-service training for clinicians to be more vigilant in diagnosing BD.

## Background

Bipolar Disorder (BD) is a serious, debilitating mental illness [1]. It affects the individual’s social, occupational and cognitive functions [2]. These negative effects not only affect the individual but his/her family as well [3, 4]. BD is a mood disorder characterized by extreme changes in one’s mood; with the mood fluctuating from extreme elation to excessive sadness. There are different types of BD, with BD I and BD II being the most common types [5]. Mania is the hallmark of BD I, while hypomania (in the absence of mania) and, at least one episode of depression, define BD II [5]. Despite being common, BD I and BD II can be misdiagnosed. BD I may be misdiagnosed as a psychotic disorder such as schizoaffective or schizophrenia due to the presence of psychotic features. While BD II has often been misdiagnosed as major depressive disorder [6, 7]. Due to these variations in presentation, some patients have received ineffective treatment leading to poor outcomes [6–8].

Globally, there are over 45 million people living with BD with a noted increase in its prevalence over the past 2 decades [9]. The global lifetime prevalence for BD is estimated to be between 1–2% [5]. However, studies have noted some cross-national differences in the prevalence ranging between 0.1% to 4.4% [12–14]. The burden of BD among hospitalized participants is even higher ranging between 30.7% - 60.0%, the highest being among those living with HIV [10]. In Zimbabwe, a study looking at the prevalence of HIV and its associated factors conducted at psychiatric out-patients clinics showed 22.2% of outpatients had a documented diagnosis of BD [11]. This is the prevalence rate which might reflect the burden of BD in the Zimbabwean clinical setting given there is no other published data on prevalence or associated factors of BD in Zimbabwe.

BD has been associated with varying demographic factors, clinical characteristics, and comorbidities which may differ according to regions [12]. There have been contrasting results in its association with gender and socioeconomic status [2, 10, 13, 14]. Three quarters of individuals with BD have a lifetime risk of a comorbid mental illness such as anxiety disorders and alcohol and substance use disorders. The presence of these comorbid mental and medical conditions such as HIV have been associated with poor treatment responses, poor quality of life and increased mortality [15–17].

Despite the high prevalence of BD and known negative effects, BD remains underdiagnosed and little is known about its burdens or associated factors in Zimbabwe. This study therefore aimed to determine the prevalence and associated factors of BD among hospitalized patients at psychiatric hospitals in Zimbabwe.

## Materials and methods

### Study design and setting

The quantitative cross-sectional study was carried out at three tertiary psychiatry hospitals in Zimbabwe: Harare (now Sally Mugabe), Parirenyatwa and Ingutsheni Psychiatric Hospitals. These are public/government referral hospitals attending to patients from district hospitals, primary care clinics, private practitioners, and other disciplines within the Zimbabwean referral pathway of the health system. The three hospitals offer emergency services, inpatient and outpatient services as well as psychosocial services [18].

### Study sample

The sample of 272 participants was drawn from both acute/admissions and subacute wards for adult male and female participants. Participants who were over the age of 18 years and able to give verbal and written consent were included. Those who had visible/obvious mental incapacitation because of mental retardation/intellectual disability or severe dementia were excluded. The patients who were sedated, frankly psychotic or were in seclusion were reassessed for eligibility later. It was necessary to reassess these patients so that those who might have mania presenting with psychotic features were not missed.

A pilot study with thirty participants, who were also included in the total study sample, was conducted to assess study feasibility. An estimated prevalence of 23% obtained from the pilot study was used to calculate the sample size. Given the prevalence of BD of 23% from the pilot study, a sample size of 272 was calculated using the Dobson’s formula [19]. Prospective participants, who met the inclusion criteria, were consecutively selected into the study.

### Data collection instruments

1. A socio-demographic questionnaire, designed for this study was used to collect participants’ information such as their age, level of education, occupation, their medical history including HIV status as well as history of alcohol and substance use. The participants who did not know their HIV status were tested if they were willing.
2. A diagnosis of BD I or BD II was made using the ‘Mini International Neuropsychiatric Interview (M.I.N.I) for mania-hypomania and major depression.’ The M.I.N.I is a screening tool with good psychometric properties as compared to the Structured Clinical Interview for DSM (SCID) diagnosis which is considered gold standard. The M.I.N.I has been validated and adapted in several countries but not in Zimbabwe [20].
3. The Alcohol Use Disorders Identification Test (AUDIT) and the Drug Use Disorders Identification Test (DUDIT) were used to make a probable diagnosis of Alcohol and Substance Use Disorders. The AUDIT/DUDIT is a 10-item screening tool developed by the World Health Organisation to identify harmful or hazardous use of alcohol and other substances. The tool has been validated in several countries and translated to other languages other than English [21–23]. The tool has not been validated in Zimbabwe.

### Data collection procedures

Data was collected between January 2020 and March 2020. Participants were assessed for eligibility through the admissions register. Prospective participants were identified and approached in the presence of their guardians to obtain informed consent to participate in the study.

After the informed consent process, the participants answered the study socio-demographic questionnaire, M.I.N.I and AUDIT/DUDIT. A diagnosis of BD was made according to the M.I.N.I. The substance misuse was ascertained using the AUDIT and DUDIT.

**Ethics approval and consent to participate.** Ethical approval was obtained from the Joint Research Ethics Committee for the University of Zimbabwe and Parirenyatwa Group of Hospitals (JREC), Harare (Sally Mugabe) Hospital and Ingutsheni Hospital Ethics Committees and Medical Research Council of Zimbabwe (MRCZ) in accordance with 1964 Helsinki Declaration.

Informed consent was sought before enrolling participants. Consent forms were available in English and the local languages, Shona and Ndebele, translated version. Participants were free to opt out at any point of the study without them incurring any penalties. Quality of care was not compromised in individuals who chose not to participate in the study.

### Data analysis

Data was entered into Excel and then exported to STATA S/E13.0 for data management and analysis. Categorical variables were expressed as frequencies (percentages). Continuous variables were summarised as means and standard deviations if normally distributed and as medians and interquartile range if the continuous variables were not normally distributed. Univariate logistic regression was performed to identify predictors for BD. All 95% confidence intervals (CI) which excluded 1 or equivalently with p<0.05 were regarded to be statistically significant. Multivariate logistic regression was performed to assess the effects predictors on BD. All statistically significant variables from the univariate analysis were considered as potential candidates for the multivariate logistic regression model.

## Results

Table 1 shows the participants’ characteristics. A total of 272 participants were interviewed. Their age ranged from 18–75 years (mean 34.8±10. 2). Almost half of the participants (N = 151) were male. Half of the participants (N = 137) had primary school level of education or below with almost two thirds (N = 163) being unemployed. A third (N = 84) of the participants had been admitted for the first into the psychiatric hospital. One-fifth (N = 55) had been previously diagnosed with BD. About three-quarters (N = 202) were HIV negative. Less than a fifth (N = 44) of participants had comorbid conditions they were living with. About two-fifths (N = 123) had a history of defaulting their medications and another two-thirds (N = 109) had a history of substance abuse.

**Table 1:** Characteristics of study participants.

Participant’s Characteristics	Frequency (%)
Gender	
Male	151 (55.5%)
Female	121 (44.5%)
Marital status	
Single	160 (58.8%)
Married	61 (22.4%)
Other	51 (18.8%)
Race	
Black African	271 (99.6%)
Mixed race	1 (0.4%)
Employment	
Not employed	163 (59.9%)
Formal	40 (14.7%)
Informal	69 (25.4%)
Income	
≤US$168.00	255 (93.75%)
>US$168.00	17 (6.25%)
Educational level	
Primary level and below	137 (50.4%)
Up to secondary level	127 (46.7%)
Above secondary level	8 (2.9%)
Prior admission	
No	84 (30.9%)
Yes	188 (69.1%)
Age of symptom onset	
≤21	99 (36.4%)
>21	173 (63.6%)
Prior BD diagnosis	
No	217 (79.8%)
Yes	55 (20. 2%)
History of defaulting medications	
No	123 (45.2%)
Yes	149 (54.8%)
Illicit substance use	
No	109 (40.1%)
Yes	163 (59.9%)
HIV status	
Negative	202 (74.3%)
Positive	67 (24.6%)
Not known	3 (1.1%)
Other medical conditions	
No	221 (81.3%)
Yes	44 (16. 2%)

### Prevalence of BD

A total of 107 (39.3%) of the study participants were found to have BD I based on a lifetime history of a manic episode according to M.I.N.I. Of note no participants in this study had BD II.

### Factors associated with BD

Univariate analysis showed significant odds ratio (OR), formal employment (OR = 4.46, 95%CI 2.11–9.43), a reported previous diagnosis of BD (OR = 5.34, 95%CI 2.79–10. 2), earning more than US$168 (OR = 3.03, 95%CI 1.09–8.47) and a history of defaulting medications (OR = 1.81, 95%CI 1.09–2.97) (see Table 2). On multi-variate analysis, being formally employed (AOR = 3.69, 95%CI: 1.55–8.79), a history of defaulting medications (AOR = 1.90, 95%CI: 1.02–3.57) and a reported previous diagnosis of BD (AOR = 5.66, 95%CI: 2.72–11.8) remained statistically significant (see Table 3).

**Table 2:** Univariate analysis of participants’ characteristics.

Participant’s Characteristics	Bipolar disorder		Odd Ratio (95%CI)	p-value
	No	Yes		
Gender				
Male	96 (35.3%)	55 (20. 2%)	1	
Female	69 (25.4%)	52 (19.1%)	1.32 (0.81–2.15)	0.272
Marital status				
Single	105 (38.6%)	55 (20. 2%)	1	
Married	32 (11.8%)	29 (10.7%)	1.73 (0.95–3.15)	0.073
Other	28 (10.3%)	23 (8.5%)	1.57 (0.83–2.98)	0.163
Employment				
Not employed	107 (39.3%)	56 (20.6%)	1	
Formal	12 (4.4%)	28 (10.3%)	4.46 (2.11–9.43)	0.00*
Informal	46 (16.9%)	23 (8.5%)	0.96 (0.53–1.73)	0.88
Income				
≤US$168.00	159 (58.5%)	96 (35.3%)	1	
>US$168.00	6 (2.2%)	11 (4.0%)	3.03(1.09–8.47)	0.03*
Educational level				
Primary level and below	86 (31.6%)	51 (18.8%)	1	
Up to secondary level	74 (27.2%)	53 (19.5%)	1.21 (0.74–1.98)	0.454
Above secondary level	5 (1.9%)	3 (1.1%)	1.01 (0.23–4.41)	0.988
Age of symptom onset				
≤21	55 (20. 2%)	44 (16. 2%)	1	
>21	110 (40.4%)	63 (23. 2%)	0.72 (0.43–1.18)	0.193
Prior admission				
No	50 (18.4%)	34 (16. 2%)	1	
Yes	115 (42.3%)	73 (26.8%)	0.93 (0.55–1.58)	0.797
Prior BD diagnosis				
No	149 (54.8%)	68 (25.0%)	1	
Yes	16(5.9%)	39(14.4%)	5.34 (2.79–10. 2)	0.00*
History of defaulting medications				
No	84 (30.9%)	39 (14.3%)	1	
Yes	81 (29.8%)	68 (25.0%)	1.81 (1.09–2.97)	0.02*
Illicit substance use				
No	67 (24.6%)	42 (15.4%)	1	
Yes	98 (36.0%)	65 (23.9%)	1.06 (0.64–1.74)	0.82
HIV status				
Negative	120 (44.1%)	82 (30.2%)	1	
Positive	43 (15.8%)	24 (8.8%)	0.82 (1.09–1.45)	0.49
Not known	2 (0.7%)	1 (0.4%)	0.73 (0.07–8. 20)	0.80
Other medical conditions				
No	140 (51.5%)	88 (32.4%)	1	
Yes	25 (9. 2%)	19 (6.9%)	1. 21 (0.63–2.32)	0.569

*statistically significant at p≤0.05

**Table 3:** Factors associated with BD on multivariate analysis.

Participant’s Characteristics	AOR (95%CI)	p-value
Employment		
Not employed	1	
Formal	3.69 (1.55–8.79)	0.003*
Informal	0.62 (0.31–1.27)	0.193
Prior BD diagnosis		
No	1	
Yes	5.66 (2.72–11.8)	0.00*
History of defaulting medications		
No	1	
Yes	1.90 (1.02–3.57)	0.045*

*statistically significant at p≤0.05

## Discussion

The study prevalence of BD was 39.3% which is similar to the one reported in a systematic review on BD carried out on patients in psychiatric hospitals in Sub-Saharan African countries which ranged between 30.7% and 60.0% [10]. These studies were conducted in psychiatric hospitals in Sub-Saharan African countries with similar geographical, cultural, and socioeconomic statuses as Zimbabwe. However, the Madziro-Ruwizhu et al (2019) study found a prevalence of 22.2% of documented diagnosis of BD in medical records in the outpatient clinic at Harare and Parirenyatwa Psychiatric hospitals [11]. This difference might be due to the difference in methodology. Madziro-Ruwizhu et al reviewed the diagnosis of BD from the participants’ outpatient medical records. The medical records diagnosis is based on clinical assessment, whereas the MINI is a BD screening tool used in this study may overestimate the diagnosis of BD.

All the participants who were found to have BD in this study had BD type I. This is similar to other studies done in various regions in Africa [10]. BD type I is characterised by at least one lifetime episode of mania which is regarded as the most severe type of BD which requires hospitalisation. The other milder types of BD are usually managed in the outpatient clinic or go undiagnosed in the community [24]. Participants are also more likely to seek medical attention for the more distressing symptoms of mania at a tertiary hospital [24].Patients with BD are also more likely to seek medical attention for the more distressing symptoms of mania at a tertiary hospital [24]. BD type I patients require medications in the acute phase of illness and for prophylaxis. Unfortunately, due to economic challenges faced by LMICs like Zimbabwe, the public hospitals often do not have these essential medications. The hospital management should therefore prioritise availability of medications such as mood stabilisers and atypical antipsychotics needed for the management of BD. This will reduce hospital stay and relapse rates thereby cutting hospital costs.

A previous lifetime reported diagnosis of BD was significantly associated with a diagnosis of BD when using the M.I.N.I. The reported diagnosis was based on what the patient had been informed by their clinician at any point in their life. A study done in Norway found that clinicians in their usual care tend to underdiagnose BD as compared to when the M.I.N.I was used [25]. In a country where there are only 19 psychiatrists for a population of 15.1 million people, there is need to strengthen the capacity of other mental health clinicians [26, 27]. Mental health clinicians at tertiary mental health institutions include psychiatric nurses, junior doctors and registrars specialising in psychiatry. These professionals often work with one or two consultant psychiatrists per institution leaving them heavily involved in decisions to do with diagnosing and treating of patients. Although these professionals have received pre-service training on how to conduct structured clinical interviews and prescribe medications, continuous in-service training remains essential. In service-training on the diagnosis of BD will ensure the condition is not under recognised and is treated correctly. It is recommended that clinicians at all levels of mental health services must be vigilant in assessing for BD. The hospital management should channel medication such as mood stabilisers and atypical antipsychotics towards the management of BD. This will reduce relapses which need hospitalisation thereby cutting hospital costs as the participants do not pay for mental health government services in Zimbabwe.

In Zimbabwe, majority of the productive population is not employed and over 34% live in extreme poverty [28]. Of those that are employed, majority work in the informal sector and usually earn better than those formally employment. The monthly wages of those in the formal sector are in the local currency are often eroded in value by the time they receive their earnings due to the high inflation rates [29]. Our study showed an association between BD and formal employment. This could suggest that BD patients are only able to work in a structured environment unlike the general population. This brings concerns about the loss in functionality and low employment rates.BD patients will likely miss job opportunities in the informal sector which have higher income in economies such as that of Zimbabwe. Additionally, patients with BD may fail to keep their jobs or are being stigmatised hence not getting equal employment opportunities. Therefore, BD association has more to do with retaining employment [17, 30]. Focusing on enhancing vocational skills training for all patients with BD could help ensure they are gainfully employed particularly in the informal sector.

In keeping with literature, a history of defaulting medication was significantly associated with BD in this study. Up to 40% of patients with BD default medication during their treatment, a rate similar to that in this study [31]. Our study population had multiple factors known to be determinants of medication compliance. Factors such being of low socioeconomic status, little to no income, comorbid substance abuse and long duration of treatment were present [31, 32]. Other patient, clinician and treatment factors known to be associated with defaulting medication were not explored in this study. However, the history of defaulting medication could have been under reported making the association with BD more likely.

## Limitations

Our study only recruited participants from tertiary psychiatric hospitals. This may make it difficult to generalize our findings for patients with milder forms of BD who were not part of the study population. Consideration should also be given to the fact that recall bias might result in the under reporting of symptoms and underestimation of the prevalence of BD. Finally, despite the M.I.N.I being a screening tool with good psychometric properties, it can overestimate the prevalence of BD and it has not been validated in Zimbabwe.

## Conclusion

The prevalence of BD among admitted patients in tertiary institutions in Zimbabwe is high. Factors associated with BD among admitted patients are a previous reported diagnosis of BD, defaulting medication and being formally employed. There is need for in-service training to increase vigilance in diagnosing BD by clinicians so BD is not missed. Further research should be undertaken to identify factors associated with all BD spectrum diagnoses and to validate screening tools such as the M.I.N.I which can be used at primary care level by non-specialised clinicians.
